# Peptide Targeting of PDZ-Dependent Interactions as Pharmacological Intervention in Immune-Related Diseases

**DOI:** 10.3390/molecules26216367

**Published:** 2021-10-21

**Authors:** Luis H. Gutiérrez-González, Selma Rivas-Fuentes, Silvia Guzmán-Beltrán, Angélica Flores-Flores, Jorge Rosas-García, Teresa Santos-Mendoza

**Affiliations:** 1 Department of Virology and Mycology, Instituto Nacional de Enfermedades Respiratorias Ismael Cosío Villegas, Mexico City 14080, Mexico; lhgut@iner.gob.mx; 2 Department of Research on Biochemistry, Instituto Nacional de Enfermedades Respiratorias Ismael Cosío Villegas, Mexico City 14080, Mexico; selmarivas@gmail.com; 3 Department of Microbiology, Instituto Nacional de Enfermedades Respiratorias Ismael Cosío Villegas, Mexico City 14080, Mexico; guzman.silvia@gmail.com; 4 Laboratory of Immunopharmacology, Instituto Nacional de Enfermedades Respiratorias Ismael Cosío Villegas, Mexico City 14080, Mexico; ffa_ff@uaem.mx (A.F.-F.); jorg_rosgar@yahoo.com.mx (J.R.-G.); 5 Department of Molecular Biomedicine, Centro de Investigación y de Estudios Avanzados, Mexico City 07360, Mexico

**Keywords:** PDZ domains, PDZ-dependent interactions, peptide inhibitors, immune-related disorders

## Abstract

PDZ (postsynaptic density (PSD95), discs large (Dlg), and zonula occludens (ZO-1)-dependent interactions are widely distributed within different cell types and regulate a variety of cellular processes. To date, some of these interactions have been identified as targets of small molecules or peptides, mainly related to central nervous system disorders and cancer. Recently, the knowledge of PDZ proteins and their interactions has been extended to various cell types of the immune system, suggesting that their targeting by viral pathogens may constitute an immune evasion mechanism that favors viral replication and dissemination. Thus, the pharmacological modulation of these interactions, either with small molecules or peptides, could help in the control of some immune-related diseases. Deeper structural and functional knowledge of this kind of protein–protein interactions, especially in immune cells, will uncover novel pharmacological targets for a diversity of clinical conditions.

## 1. Introduction

Protein–protein interactions (PPIs) are physical contacts of high specificity between two or more proteins. They are the result of biochemical events caused by interactions, such as electrostatic forces, hydrogen bonding, and the hydrophobic effect; these PPIs must be mechanistically determined, and not accidental, with a non-generic interaction interface and within a biological context [1]. It has been reported that over 80% of proteins do not operate alone but in complexes that include a large family of enzymes, transcription factors, and signaling proteins, among others [2]. Most PPIs occur in a cell or in a living organism in a specific biological environment and consist of physical contacts with molecular associations between chains. There are approximately 650,000 PPIs in a human cell [3,4], of which a vast number are mediated by protein interaction modules or domains that have evolved to recognize specific elements of the partnering proteins [5]. The PPI complex network, which has been named “interactome”, plays an important role in physiological and pathological processes, including signal transduction, cell proliferation, growth, differentiation, and apoptosis, among others [6]. Canonical examples of PPI domains include Src homology 2/3 (SH2/3) domains, WW domains, phosphotyrosine-binding (PTB) domains, and homologous PDZ (PSD-95/Disc-large (DLG1)/ZO-1) domains [7].

**Figure 1:**
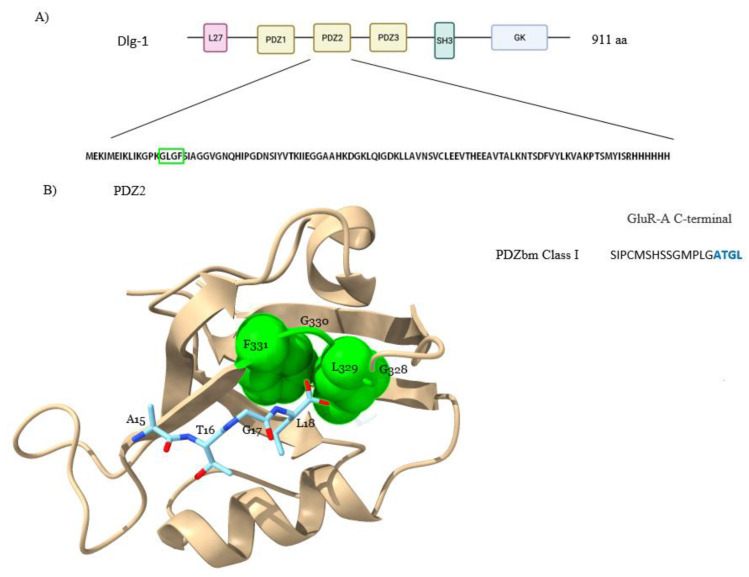
**Structure of the second PDZ Domain of Dlg1 in complex with the GluR-A C-terminal PDZbm (PDB ID: 2GLG)**. (**A**) Schematic representation of Dlg1 showing its three PDZ domains and the primary sequence of the PDZ2 domain; GLGF sequence is marked in a green box. (**B**) Three-dimensional structure of PDZ2 domain of Dlg1 (golden) in complex with the class I PDZbm of the subunit GluR-A (blue) of glutamate receptors [11]. The conserved GLGF sequence in the PDZ domain is shown in green. The C-terminal sequence of GluR-A including the PDZbm is shown (right). Figure is drawn with ChimeraX 1.2.5. (University of California at San Francisco, San Francisco, CA, USA).

The PDZ family of proteins is one of the largest in the human proteome. The name PDZ derives from the first three proteins in which these domains were identified: PSD-95 (post-synaptic density protein of 95 kDa), Dlg (Discs large protein) and ZO-1 (zonula occludens-1 protein) [8]. These proteins mainly function as molecular scaffolds and are characterized by the presence of at least one PDZ domain (frequently multiple). PDZ domains are the most common protein domains in the human genome and have an important role in the formation of complexes with specific functions [5,9,10]. A PDZ domain contains about 90 amino acids folded in a globular structure of six β-strands and two α-helices in which the conserved sequence GLGF is important for the interaction with its ligands, usually short linear amino acid (aa) sequences (SLiMs) at the C-terminus named PDZ binding motifs (PDZbm) (Figure 1).

Short linear motifs (SLiMs) are short, unstructured sections of protein sequences that mediate in PPIs [12]. SLiMs are widely used by nature to confer dynamism to protein–protein interactions and responsiveness to cellular biochemical complexes under various stimuli. PDZ domains participate in multiple PPIs related to a diversity of cellular functions and pathogen recognition, which are mostly regulated by a specific kind of SLiM: the PDZbm. This motif is defined by the four last aa and classified into three groups according to the consensus sequence: X-S/T-X-Φ for class I; X-Φ-X-Φ for class II, and X-D/E-X-Φ for class III (where X is any amino acid, and Φ is a hydrophobic amino acid).

It is currently known that the PDZ domains can also bind phospholipids and internal peptides; an example of this kind of interaction is that of the syntenin with phosphatidylinositol 4,5-bisphosphate (PIP2), which has been suggested to be of importance for targeting the protein to membrane and nuclear pools of PIP2 [13]. An example of an internal peptide binding is that of the nitric oxide synthetase (nNOS) β-hairpin structure binding to the syntrophin PDZ domain [5,14].

The PDZ-binding motifs (PDZbms) were first described around 1996 in three viral oncoproteins: human T-lymphotropic virus type 1 (HTLV-1) Tax, human adenovirus E4-ORF1, and human papillomavirus E6, all of which target Dlg1 [15,16,17]. Since then, many other viruses that encode proteins with PDZbms-targeting PDZ proteins have been found. Several pathogens possibly use PDZbms that have evolved to evade host immune response by disrupting host PDZ-dependent interactions in infected cells [18,19,20,21,22]. The study of PDZbm–PDZ domain binding in infections is an interesting subject. In this work, however, we will focus on endogenous PDZbm–PDZ protein interactions; in particular, we will discuss the possibility of designing inhibitors that, analogously to PDZbm in pathogens, could target host components involved in human immune response.

## 2. Peptide Inhibitors for Targeting PDZ Protein–Protein Interactions

Given the low specificity of current treatments for human diseases, new therapeutic targets have been proposed in recent decades. The main goal in drug design and development is to target a single biological entity with high selectivity and efficiency; it focuses on specific signaling pathways for the management of diseases at the cellular and molecular level. PDZ-dependent PPIs have been widely studied as therapeutic targets in infectious diseases [23,24,25]. For example, it has been recently demonstrated that the interaction of the PDZbm of the E protein of SAR-CoV-2 virus (DLLV) with the second PDZ domain of the human ZO-1 protein reduces the airway barrier damage and prevents viral spread [26].

The involvement of PDZ proteins in neurodegenerative and mental illnesses is well recognized. In neuronal disorders, in which a major goal is the conservation of homeostasis, PDZ–PPIs play a key role in regulating many signaling pathways. For instance, the PDZ domain of neuronal nitric oxide synthase (nNOS) regulates anxiety-associated behaviors through the interaction with the PDZbm (EIAV) of its ligand CAPON (carboxyl-terminal PDZ ligand of neuronal nitric oxide synthase protein); dissociation of this interaction results in anxiolytic-like effects in vivo in a mice model [27]. In cerebrovascular injury, the PDZbm (ITKV) of the phosphatase and tensin homolog (PTEN) induces apoptosis and axonal growth in the nervous system by its interaction with PDZ domains of MAGI-2 (membrane-associated guanylate kinase inverted-2) and MAST2 (microtubule-associated serine/threonine kinase-2), and the peptide-based interruption of these interactions have demonstrated to be an effective therapy that results in neuroprotective effects [28]. Moreover, the interaction of the PDZbm of PTEN with the PDZ domain of the kinase MAST2 blocks neuronal survival, neurite outgrowth, and regeneration; the targeting of this interaction by the viral PDZbm (QTRL) of the G protein of rabies virus (RV) promotes neuronal survival by disrupting the PTEN-MAST2 complex [29,30]. With this knowledge, a series of peptides have been designed and tested for their potential use as drugs in neurodegenerative disorders with satisfactory results [30]. The peptide NH2-SWESHKSGGQTRL-COOH (NV1), corresponding to the last 13 aa at the C-terminal sequence of the RV G protein, successfully disrupts the PTEN-MAST2 complex. Furthermore, sequence modification of NV1 derived in a new peptide NV3 with higher affinity for MAST2, thus attaining higher capacity of neurite outgrowth induction [30]. The identification of both endogenous and pathogen-derived PDZbms may shed light on important functional interactions that are useful for drug design.

In recent years, the development techniques for peptides have improved, thus increasing the number of peptides entering clinical trials [31,32]. PDZ-targeting molecules in clinical trials include NA-1 (previously known as nerinetide or TAT-NR2B9c), a neuroprotective 20 aa peptide with the last nine C-terminal residues (KLSSIESDV) of the 2B subunit of the NMDA glutamatergic receptor (GluN2B) that selectively blocks the interaction of both GluN2B and nNOS with PSD-95, thus alleviating the excitotoxic effects of excessive NO production during acute ischemic stroke. By mid-2021, NA-1 is in Phase III clinical trials [33,34] (Table 1).

PDZ-dependent interactions have been documented in a variety of diseases. In particular, blocking PDZ-dependent interactions in neurological and tumorigenic disorders has had positive therapeutic effects in the treatment of diseases, such as cancer, neuropathic pain, or ischemic stroke, and specific blockers are either commercially available or in clinical trials [33,35,38]. Thus, we can straightforwardly assume that blocking PDZ-dependent interactions, either with small molecules or with peptides, in immune-related diseases may also have important therapeutic benefits. We propose that specific peptides may inhibit the PDZ-dependent interactions in the syntenin-CD6, syntenin-IL-5Ra, and Scrib-p22phox complexes with different pharmacological effects.

The use of peptides as therapeutic targets has been extensively studied because it provides advantages, such as target specificity and high affinity. The development of these therapeutic peptides is a promising area of research that, in the near future, could provide novel and valuable treatments [6].

## 3. Role of Endogenous PDZ Interactions in Human Immune Processes

In this section, we briefly review from scattered literature some PDZ PPIs in the immune system and then propose that peptide interventions of PDZ PPIs can be applied to immune-related diseases, as they are currently being used in many neuronal disorders and cancer. We propose specific peptides with inhibiting effects on PDZ-dependent interactions to be evaluated as potential therapeutic agents in diseases, such as severe asthma, autoimmune diseases, or hyperinflammation.

### 3.1. G Protein-Coupled Receptors (GPCRs)

G protein-coupled receptors (GPCRs) are involved in the regulation of many aspects of human physiology and have been considered pharmacological targets for several diseases, such as metabolic disease [50], disorders of calcium homeostasis [51], or breast cancer [52]. Around 20% of human GPCRs bear a PDZbm, and PDZ proteins play a key role in the regulation of GPCR subcellular localization, signal transduction, trafficking, and recycling [53,54]. Targeting of PDZ-dependent interactions of these receptors may lead to specific therapeutic strategies.

#### 3.1.1. Chemokine Receptors

Chemokine receptors belong to the large family of GPCRs, with many of them containing PDZbms. Chemokine receptors orchestrate leukocyte trafficking in homeostasis and in response to stimuli. Upon binding their ligands, these receptors activate downstream signaling pathways leading to functional responses until they are desensitized and are often endocytosed for subsequent recycling [53].

CXCR2 is a chemokine receptor that binds the chemokines CXCL1-3 and CXCL5-8 [55]; its activation has been linked primarily to the recruitment of neutrophils, specialized innate immune cells involved in the first line of defense against pathogens due to their high motility and bactericidal activity. Nevertheless, an excessive neutrophil recruitment or activity may lead to extensive release of toxic agents and tissue damage, thus exacerbating inflammation with deleterious outcomes, as has been observed in severe COVID-19 [56,57]. Additionally, neutrophils may also take part in pathological conditions, such as tumor cell proliferation and angiogenesis that supports tumor growth [58].

**Figure 2:**
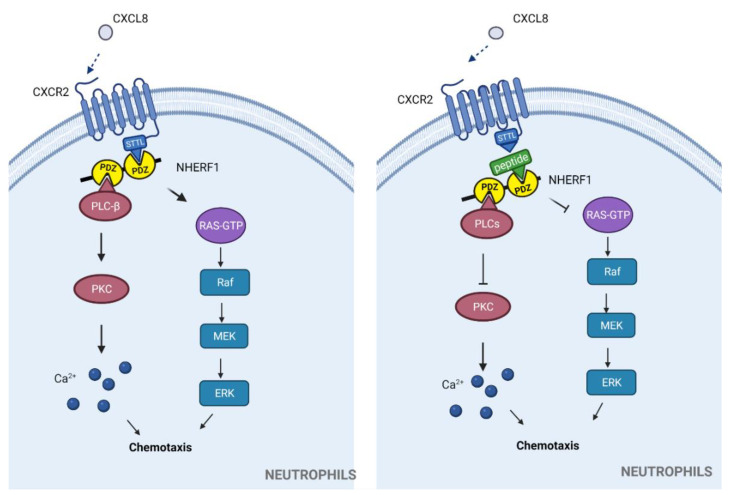
**PDZ-dependent interaction of CXCR2 with NHERF1 is required for neutrophil functions.** In response to the chemokine CXCL8, NHERF1 organizes a tripartite complex through recognition of the PDZbm STTL of CXCR2 and PLC-β, whose downstream signaling leads to neutrophil calcium mobilization and chemotaxis (**left**). Disruption of this complex by exogenously added peptide corresponding to the last 13 aa of CXCR2 inhibits these neutrophil functions (**right**).

CXCR2 contains a class I PDZbm (STTL), which is able to bind to the PDZ domain 1 of the Na(+)/H(+) exchanger regulatory factor 1 (NHERF1) [59]. This PDZ-dependent interaction appears to be involved in the stability/recycling of the receptor. A mutant CXCR2 lacking the PDZbm, ectopically expressed in HEK 293 cells, resulted in increased CXCR2 lysosomal localization and receptor degradation with concomitant reduction in chemotaxis towards CXCL8, compared to WT CXCR2 expression [60]. NHERF1 is a scaffold protein containing two PDZ domains in tandem through which it is able to assemble a tripartite regulatory complex coupling CXCR2 with its downstream effector phospholipase C (PLC)-β2. These PDZ-dependent interactions are indispensable to induce calcium mobilization, chemotaxis, and transmigration, being thus crucial for neutrophil functions [39]. Of note, an exogenously added synthetic peptide corresponding to the last 13 aa of CXCR2 (FVGSSSGHTSTTL), which include the PDZbm, is able to disrupt endogenous PDZ-dependent CXCR2 interaction with NHERF1 and PLC-β2 complex, inhibiting calcium mobilization and chemotaxis in neutrophils upon CXCR2-ligand stimulation [39] (Figure 2). Through an analogous process, the same peptide can inhibit cell proliferation and invasion in pancreatic cancer cell lines (HPAC, Colo357 and HPDE) in response to the CXCR2 chemokine ligand. Additionally, in an in vivo tumorigenesis model, this peptide also inhibits tumor growth [61].

The homing of circulating endothelial progenitor cells (EPC), which contribute to the formation of new blood vessels in the adult organism, is also regulated by CXCR2 [62]. Tube formation experiments demonstrated that murine EPCs co-cultured with human dermal microvascular endothelial cells (HDMVECs) partially fail to incorporate into the tube when cultured with the C-terminal peptide of CXCR2. Similar results were obtained in in vitro adhesion and migration assays, while a more marked inhibition was found in extracellular calcium mobilization experiments [62].

Thus, the PDZbm-dependent interactions of CXCR2 regulate its functional responses and different aspects of its biology, including receptor recycling, calcium influx, chemotaxis, cell recruitment, cell proliferation, and angiogenesis, making these CXCR2 interactions a potential pharmacological target. CXCR2 antagonists could modulate acute and chronic inflammation in various pathologies, and some are currently being tested in clinical studies for chronic diseases, such as chronic obstructive pulmonary disease, asthma, cystic fibrosis, and cancer [63,64].

Proinflammatory cytokines, such as IL-1β, IL-6, IL-8, and GM–CSF, which are produced by infiltrating monocytes and neutrophils, cause significant damage to lung tissue in severe COVID-19 [65]. The use of CXCR2 inhibitors has been proposed to be useful for downregulating monocyte and neutrophil recruitment and activation in COVID-19 [66]. Here, we propose PDZbm-dependent CXCR2 interactions as a novel bona fide inhibitory target.

#### 3.1.2. Other Chemokine Receptors with PDZbms

In addition to CXCR2, several human chemokine receptors belonging to three chemokine receptor families (CXC, CC, and CX3C) contain PDZbms in their cytoplasmic tails: CXCR1, CXCR3, CXCR5, CXCR6, CCR3, CCR5, CCR6, CCR9, CCR11, and CX3CR1 [67]. For GPCRs in general, PDZbms are involved in different functions, such as the increase in calcium influx, desensitization, activation of the inositol 1,4,5-trisphosphate pathway, ERK, PLC, and small GTPases, such as Rho activation [53]. Which of these functions are regulated by PDZbms in chemokine receptors remains to be determined, but we can speculate that they might be novel targets for treating immunological disorders.

Given that most chemokine receptors are promiscuous, CCR6, and CX3CR1 receptors, which bind to a single ligand (CCL20 and CX3CL1, respectively), may be useful as a model for the study of PDZ targeting by therapeutic inhibitors. CCR6 is abundantly expressed by immunomodulatory T cell subsets, such as regulatory T cells (Treg) and IL-17-producing T cells (Th17) [68]. In a rheumatoid arthritis model, it was shown that CCR6 and CCL20 expressions are necessary for the migration of these Th17 cells, whose presence in the joints favors disease progression [69]. The CCL20–CCR6 axis is also involved in the pathophysiology of several types of cancer, for example, in gastric, prostate, and lung cancer; thus, this molecular axis could be contributing to tumor growth, dissemination of neoplastic cells, and colonization of organs different from where the primary tumor was generated [70]. On the other hand, CX3CR1 appears to be involved in different functions, such as proliferation, cell adhesion, and migration, which contribute to tumorigenesis and cancer progression in gastric, pancreatic, or lung cancers. Such functions of CX3CR1 have been observed not only in cancerous cells themselves but also in immune cells, such as macrophages that in turn synergize in cancer promotion [71]. In fact, motility and metastasis of pancreatic adenocarcinoma cells can be inhibited with the CX3CR1 specific inhibitor JMS-17-2 [71]. In addition, in acute autoimmune kidney disease and in chronic kidney disease (CKD), CX3CR1 appears to be of major importance in the recruitment of immune cells and in the processes leading to macrophage-mediated fibrosis of the kidney [72]. Some small molecules and specific CX3CR1-inhibiting antibodies are being assayed in animal models and clinical trials to treat multiple sclerosis, inflammatory bowel disease, rheumatoid arthritis, or CKD [72]. This knowledge reinforces the idea that targeting of CCR6 and CX3CR1 PDZ-interactions to modulate such immune-related disorders is an interesting topic to be explored.

### 3.2. Syntenin

Syntenin is a PDZ protein originally identified as a melanoma differentiation-associated gene (mda-9); the first function described for syntenin was as an adaptor protein involved in the trafficking of the syndecan receptor to the plasma membrane [73,74,75]. Syntenin contains an N-terminal domain (NTD) of 113 aa, two PDZ domains in tandem, and a short C-terminal domain (24 aa) with no enzymatic activity; thus, it mainly functions as scaffold protein [76]. To date, several interacting proteins for syntenin have been described as well as some functions for these interactions, including receptor membrane targeting and recycling of different receptors, the stabilization of transmembrane proteins at cell surface or their internalization, and promotion of cell migration, among others [76,77,78]. Syntenin is highly expressed and appears to be involved in the metastasis of several tumors, such as melanoma, neuroblastoma (NB), breast cancer, and small cell lung cancer, among others, by regulating cell membrane motility and migration [40,79]. Furthermore, knockdown of syntenin resulted in a reduction in NB invasion, migration, and metastasis both in vitro and in vivo [78]. It has been described that the scaffolding function of syntenin defines the molecular mechanisms of cell transformation and metastasis in NB by activation of the alpha-6 beta-4 integrin, Src kinase, and cytoskeletal regulators Rho, Rac, and Cdc42 [77,78]. With this knowledge, syntenin has become an attractive drug target for cancer treatment; both small molecules and inhibitory peptides have been developed recently for this purpose [41,80]. PDZ1i is a small molecule which binds to the first PDZ domain of syntenin, thus blocking its PDZ-dependent PPIs. PDZ1i has demonstrated an inhibitory effect on metastasis of a variety of cancers, such as glioblastoma multiforme (GBM), NB, and prostate cancer in vitro and in vivo [81,82]. On the other hand, a dipeptide designed to exert higher affinity for both PDZ domains of syntenin than endogenous ligands has also been shown to be effective in blocking cell migration in the human cancer cell line MDA-MB-435 [40] (Table 1).

In addition to its well-documented function in cancer and metastasis, syntenin has relevant functions modulating the innate and adaptive immune cells. Syntenin is involved in the induction of PD-L1 expression in breast cancer cells which in turn evade the immune response of CD8+PD1+ T lymphocytes [83]. In this manner, syntenin is able to abrogate CD8+ T cell responses; as a consequence, the inhibition of syntenin might rescue T cell cytotoxicity.

**Figure 3:**
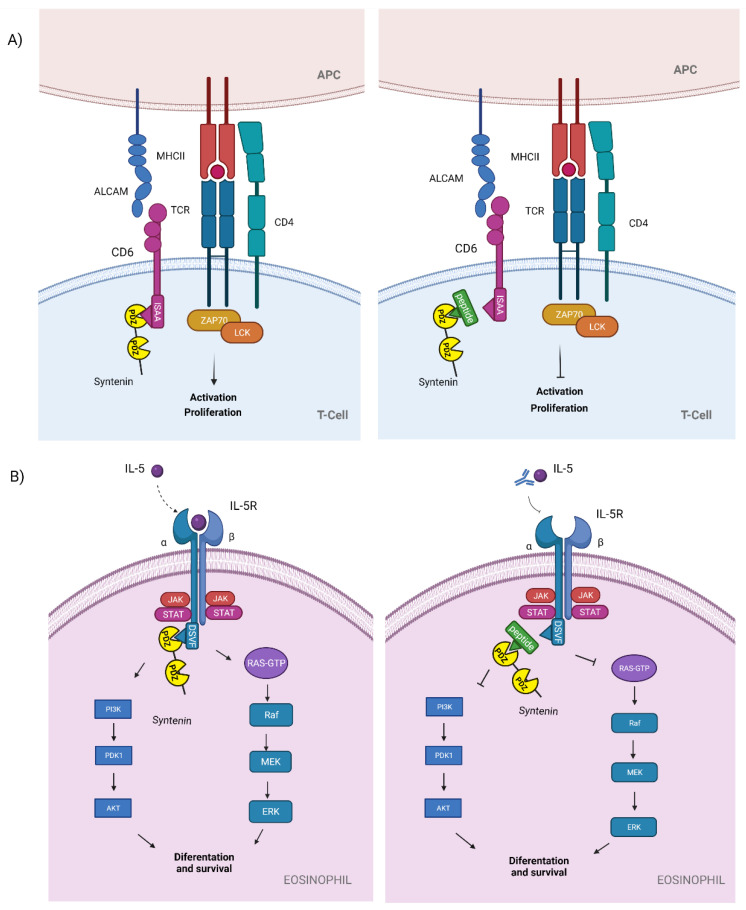
**PDZbms associated with syntenin interactions.** (**A**) The scavenger receptor cysteine-rich superfamily member CD6 molecule, expressed on T cells, recognizes ALCAM on APCs, stabilizing the immunological synapses, favoring T cell activation and proliferation through a PDZbm (ISAA) binding with a PDZ domain of syntenin (**left**). Blocking these PDZ-dependent interactions with specific peptides may inhibit Tcell activation and proliferation (**right**). (**B**) Recognition of the cytokine IL5 by IL-5R induces the interaction between the PDZbm DSVF and syntenin, leading to eosinophil differentiation and survival (**left**). Eosinophil survival can be blocked by antibodies against IL-5 and possibly by a specific peptide that interferes with the interaction between DSVF and syntenin (**right**).

Syntenin controls Rac-1 activation and actin polymerization during migration induced by CXCL12 chemotactic stimuli and during Ag recognition in T cells, as a downstream effect of syntenin phosphorylation by Src kinase; consequently, syntenin is required for the onset of T cell responses [84]. An additional pathway that contributes to T cell activation is derived from the scavenger receptor cysteine-rich superfamily member CD6, a transmembrane glycoprotein partly associated with TCR in resting conditions and recruited to the immunological synapse (IS) during Ag recognition, favoring IS maturation and T cell activation and proliferation [85]. The cytoplasmic tail of CD6 contains several consensus motifs, including phosphorylatable residues and protein interacting motifs with a class I PDZbm (ISAA). CD6 interaction with syntenin drives downstream signaling pathways in response to CD6 stimulation [85,86]. The interaction of CD6 with its ligand activated leukocyte cell adhesion molecule (ALCAM) stabilizes the immune synapse, supporting T cell activation and proliferation [85,86] (Figure 3A). CD6 has been implicated in the development of autoimmune diseases, such as multiple sclerosis (MS), and the targeting of this molecule has been proposed to improve clinical manifestations of these patients [42]. There are at least three anti-CD6 antibodies used to ameliorate experimental autoimmune encephalomyelitis (EAE) in mice and to treat additional autoimmune diseases, such as rheumatoid arthritis or psoriasis, with satisfactory results in clinical trials [43]. More investigation is needed to dissect the specific mechanisms elicited by CD6 through its PDZ-dependent interaction with syntenin, but the pharmacological interference of this interaction would probably help in the regulation of CD6-related immune responses.

IL-5 receptor is a heterodimer formed by the specific α chain (IL5Ra) and the β chain, which is shared by other two cytokine receptors, IL-3 and GM-CSF. IL5Ra contains a class-I PDZbm (DSVF) that is able to bind both PDZ domains of syntenin with higher affinity for the first one [5]. This interaction is needed for the direct activation of the transcription factor SOX4, which is recruited by the NTD of syntenin, upon IL-5 stimulation, with implications in B cell development [44]. Furthermore, the specific interaction of syntenin with IL-5Ra appears to be the driving force of eosinophil differentiation and survival by a mechanism depending on JAK/STAT and ERK1/2 phosphorylation and activation [45,87]. Allergic asthma is a chronic disease characterized by airway inflammation and hyper-responsiveness, bronchoconstriction, and muscle hypertrophy, among other specific features elicited by allergens [88,89]. Eosinophils are of major relevance in the development of airway inflammation and tissue damage during allergic asthma; severe disease is normally associated with high eosinophilia compared with non-severe asthma [45]. Of note, in such patients, exposure to allergens has been associated with increased IL-5R+ progenitor bone marrow cells and with allergic severity [45]. Nowadays, therapy with blocking antibodies against IL-5 (Mepolizumab) or IL-5Ra (Benralizumab) has proven to be effective in the treatment of severe asthma [87,90]. The use of syntenin inhibitors has been proposed to alleviate diverse allergic manifestations [45]; we now propose, specifically, the PDZ-dependent interaction of syntenin with IL-5Ra as a candidate target for such aim (Figure 3B).

### 3.3. NADPH Oxidase

The NADPH oxidases or Nox enzymes produce reactive oxygen species (ROS) as their primary and sole function. This family consists of seven isoforms: Nox1, Nox2, Nox3, Nox4, Nox5, and the dual oxidases Duox1 and Duox2. All isoforms are integral membrane proteins and transfer electrons from NADPH to O_2_, thereby generating O_2_·^−^. The Nox1-4 depend on a common subunit p22phox, while Nox5, Duox1, and Duox2 are independent of p22phox [91].

Recently, it has been demonstrated that, in macrophages, the PDZ protein Scribble (Scrib) interacts directly with the NADPH oxidase (Nox) complex in a PDZ-dependent manner, binding the class III PDZbm (DEVV) present in the p22phox subunit. Therefore, Scrib is a member of the Nox1-4-complex isoforms and is required to generate reactive oxygen species (ROS) induced by Nox complexes with a direct impact in the capacity of macrophages to destroy microorganisms [46].

Nox enzymes have several roles in many organisms from organ to cellular level, including antimicrobial defense, vasoregulation, hormone synthesis, and regulation of gene expression, cell proliferation, and differentiation [92,93,94]. For example, Nox2 produces exacerbated amounts of ROS for the antimicrobial activity of professional phagocytes, such as neutrophils, macrophages, and dendritic cells [95]. On the other hand, Nox1, 3, 4, 5, and Duox1 and 2 regularly produce smaller amounts of ROS for regulation of signaling pathways or during anabolic processes, such as endothelial vasoregulation [96,97,98,99].

These enzymes play critical roles in multiple diseases associated with oxidative stress, such as hypertension, diabetes, Alzheimer’s disease, and pre-eclampsia [93]. Therefore, Nox enzymes are considered prime target candidates for the treatment of these diseases. Diverse compounds have been postulated as NADPH oxidase inhibitors in different pathologies [100].

Hypertension is a cause of cardiovascular disease and premature death in the world. This disease is associated with oxidative stress and perivascular inflammation, critical contributors to perivascular fibrosis and accelerated vascular aging [101,102]. The oxidative stress is generated mainly for the Nox enzymes. They are expressed throughout the vessel wall, including endothelial cells, vascular smooth muscle cells, fibroblasts, perivascular adipocytes, as well as infiltrating leukocytes and increasing vascular resistance, fibrosis, and endothelial dysfunction [103,104]. It has been demonstrated in the participation of Nox1 and 2 in a hypertension experimental model [105].

Patients with type 2 diabetes mellitus (DM2) exhibit increased oxidative stress in the vasculature, which is thought to play an important role in the initiation and progression of diabetic complications, including atherosclerosis, that results in risk for stroke myocardial infarction and death [47,106]. Furthermore, Nox isoforms are upregulated in hyperglycemia, inducing diabetic cardiovascular disorders in DM2 patients [107]. Several Nox isoforms are present in the vasculature: Nox1, Nox2, Nox4, and Nox5. They probably play an important role in vascular pathobiology, inducing both inflammation and fibrosis [108].

Alzheimer‘s disease is an irreversible, progressive neuropathological disorder. There is evidence for a role of microglial Nox2 in inflammatory neurodegeneration [109]. Several studies have shown a role of microglia, the central nervous system resident macrophages, in amyloid precursor protein (APP)-dependent neurodegeneration [48,104,110,111]. APP fragments released from neurons activate Nox2 in neighboring microglia cells, which in turn exacerbate ROS production that leads to death of neighboring neurons [112]. Furthermore, Nox2 (PHOX-/-)-deficient mice are protected in a 1-methyl-4-phenyl-1,2,3,6-tetrahydropyridine (MPTP) model of Parkinson’s disease [49].

Pre-eclampsia is a pregnancy complication characterized by high blood pressure and signs of damage to other organs, most often liver and kidneys. The pre-eclamptic placenta is usually hypoxic and stimulates the release of a large number of microparticles [113]. Placental hypoxia and microparticles could stimulate the production of damage-associated molecular patterns, which activate neutrophils and dendritic cells. This activation could produce proinflammatory cytokines, including tumor necrosis factor α (TNFα) and ROS-generating NADPH oxidase, contributing to pathogenesis [114]. A mice model of pre-eclampsia demonstrated that Nox2 is central to the production of superoxide and reduced nitric oxide (NO) bioavailability that leads to endothelial dysfunction [115]. The inhibition of Nox2 by apocynin (a Nox inhibitor) in spontaneously hypertensive rats decreases vascular ROS levels, increases NO bioavailability, and lowers blood pressure [113,116].

Diverse molecules have been used in in vitro and in vivo studies that may be useful for current therapy to mitigate the adverse effects associated with disease. The first inhibitors used, such as apocynin and diphenylene iodonium, are unspecific and not isoform-selective; however, recent inhibitors are more specific and can potentially be used in some pathologies. For example, GKT137831 inhibits the Nox1, Nox4, and Nox5; ML171 inhibits Nox1, Nox2, and Nox4, and VAS2870 inhibits Nox2 [100].

**Figure 4:**
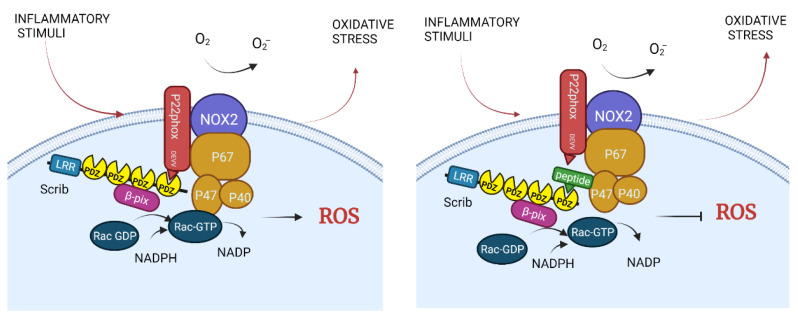
**NADPH oxidase (Nox) complex activation depends on a PDZ interaction.** The PDZ protein Scrib assembles the NADPH oxidase (Nox) complex through the interaction with the PDZbm (DEVV) in the p22phox subunit, producing ROS (**left**). The ROS production could be inhibited with a peptide that blocks the binding of DEVV with the PDZ4 domain of Scrib (**right**).

By inhibiting Scrib PDZ-dependent interactions with specific peptides, the Nox complex assembly could be blocked and thereby exacerbated ROS production could be temporarily inhibited (Figure 4). As mentioned previously, the p22phox subunit of NADPH oxidase binds to the PDZ4 domain of Scrib through a class III PDZbm (DEVV), indispensable for Nox complex assembly [46]. Moreover, additional NADPH oxidase subunits appear to have PDZbms, such as p47phox, with a class I sequence ASAV, and Nox2, with a class III sequence KENF. In fact, PDZ-dependent interactions of p47phox with NHERF1 have been described [117]. This would suggest that other PDZ-dependent interactions might be involved in ROS production; thus, it is reasonable to suppose that additional peptides or small molecules with therapeutic potential are yet to be explored (Table 1).

## 4. Concluding Remarks

In recent years, significant technological advances to design and develop specific synthetic peptides have occurred, such as in the case of peptidomimetics [23]. This has stimulated pharmaceutical industries and researchers to identify peptides of clinical utility. Viral pathogens have taught us that PDZ PPIs can be targeted to modulate cellular behavior by mimicking endogenous ligands. Thus, PDZ PPIs have been extensively studied as potential targets of small molecules and peptides as therapeutic agents. To date, there is a peptide commercially approved to treat cancer and some others are currently in clinical trials (phase II and III) to be used in neuropathic diseases. Here, we have proposed the use of peptide inhibitors of PDZ PPIs in immune-related diseases. PDZ proteins are highly conserved evolutionary and the recent recognition of several functions of some of these proteins in immune cells may open novel research fields; in particular, it remains to be established which additional PDZ proteins are expressed in immune cells and which their functions are. On the other hand, the description of PDZ-dependent interactions in immune functions and their possible involvement in immune-related diseases may allow us to consider them as good targets for novel immunotherapies.

## Figures and Tables

**Table 1:** Blockers of PDZ-dependent interactions with therapeutic applications.

Peptide/Inhibitor	PDZ-Dependent Interaction (PDZbm)	Function of the PDZ-Dependent Interaction	Inhibitor Function	Study Phase	Disease	Reference
Peptide Pen-N3 *	DVL2-PDZ: Frizzled (Internal PDZbm) (KWYGW)	DVL facilitates Wnt signal, which leads to activation of b-catenin and T cell factor (TCF)-dependent transcription of developmental genes and genes associated with tumorigenesis	Inhibits canonical Wnt	Commercial: MERCK	Cancer	[35]
Tat-P4-(C5)2 *	PICK1-PDZ1: GluA2 (HWLKV)	PICK1 regulates PKC-dependent phosphorylation of S880 of AMPAR Glu A2 subunit in trafficking and plasticity	Interferes with excessive glutamate receptor transmission in pain; disrupts the interaction of PICK1 with AMPARs	Preclinic, in vivo	Neuropathic pain	[36]
3-hydroxymethylindole **	MAGI3-PDZ2: PTEN (YKQTSV)	Prevents PTEN recruitment to the plasma membrane and allows full activation of PKB	Blocks tumorigenic processes	Preclinic, in vitro	Cancer	[37]
NA-1 *	PDS-95-PDZ1: GluN2B (tSXV)	GluN2B activates nNOs in association with PSD95, promoting excitotoxicity in ischemic stroke	Inhibits neuronal excitotoxicity; penetrates the blood–brain barrier and interferes with GluN2B intersctions	Clinic, phase III	Ischemic stroke	[33,38]
Synthetic peptide: FVGSSSGHTSTTL *	NHERF1-CXCR2 (STTL)	Neutrophil migration in exacerbated inflammation	Proposed to be used in exacerbated inflammatory-related diseases	In vitro	Excessive neutrophil recruitment, tumorigenesis.	[39]
PDZ1i ** (CGSDKEϕϕV)2 *	Syntenin-CD6 (ISAA)	T cell activation and proliferation in autoimmune diseases: MS, EAE	Proposed to be used in autoimmune diseases	Theoretical	Autoimmune diseases	[40,41,42,43]
PDZ1i ** (CGSDKEϕϕV)2 *	Syntenin-IL-5Ra (DSVF)	Eosinophil differentiation and survival in severe asthma	Proposed to be used in severe eosinophilic asthma	Theoretical	Severe eosinophilic asthma	[40,41,44,45]
To be designed. There is an inhibitor for PDZ1 and 3 domains but not for PDZ4.	Scrib-p22phox (DEVV)	ROS production in macrophages to destroy pathogens; probably exacerbated hypertension, Alzheimer’s and Parkinson’s diseases	Proposed to be used in hypertension, Alzheimer’s and Parkinson’s diseases	Theoretical	Hypertension, Alzheimer’s and Parkinson’s diseases	[46,47,48,49]

* Peptide; ** Small molecule.

## Data Availability

No new data were created or analyzed in this study. Data sharing is not applicable to this article.
